# Efficacy and safety of non-vitamin K-antagonist oral anticoagulants for retinal vascular diseases in patients with atrial fibrillation: Korean cohort study

**DOI:** 10.1038/s41598-020-61609-8

**Published:** 2020-03-12

**Authors:** Se-Jun Park, Eunyoung Lee, Kihwang Lee, Bumhee Park, Yoo-Ri Chung

**Affiliations:** 1 Division of Cardiology, Department of Internal Medicine, Gangneung Asan Hospital, University of Ulsan College of Medicine, Gangneung, Korea; 2 Department of Biomedical Informatics, Ajou University School of Medicine, Suwon, Korea; 3 Office of Biostatistics, Ajou Research Institute for Innovative Medicine, Ajou University School of Medicine, Suwon, Korea; 4 Department of Ophthalmology, Ajou University School of Medicine, Suwon, Korea

**Keywords:** Atrial fibrillation, Retinal diseases

## Abstract

We investigated the prevalence of retinal vascular occlusion and intraocular bleeding and compare their risks in patients undergoing anticoagulant therapy, either with non-vitamin K-antagonist oral anticoagulants (NOAC) or warfarin. We performed a cohort study (January 2015 to April 2018) in 281,970 patients with nonvalvular atrial fibrillation (AF) using health claims in the nationwide database of the Health Insurance Review and Assessment service of Korea. A Cox-proportional hazard regression was used to calculate the hazard ratio (HR) for retinal vascular occlusion or intraocular bleeding. The HR of retinal vascular occlusion was estimated to 1.59 (95% confidence interval [CI], 1.35–1.86) for NOAC users compared to that with warfarin users. Among the various types of NOACs, all NOACs showed higher risk of retinal vascular occlusion than did warfarin. For intraocular bleeding, the HR was estimated to be 0.86 (95% CI, 0.75–0.98) for NOAC users compared with that with warfarin users. The risk of retinal vascular occlusion was higher in NOAC users than in warfarin users, while the risk of intraocular bleeding was lower with NOAC therapy. NOACs were not found to be as effective as warfarin for retinal vascular occlusion, but safe in terms of intraocular bleeding.

## Introduction

Non-valvular atrial fibrillation (AF) is a global health burden and the prevention of AF-related thromboembolic events is a major concern^1^. Warfarin can effectively prevent stroke and systemic embolism in patients with AF but increases the incidence of major bleeding such as intracranial hemorrhage^2^. Non-vitamin K-antagonist oral anticoagulants (NOACs) have been recently approved for prevention of stroke in patients with non-valvular AF, and do not require anticoagulation monitoring^3^. Since NOACs are at least as effective and safe compared to warfarin, they have become essential for patients with non-valvular AF^4^.

Retinal vascular occlusion is a major vascular disease of the retina that can lead to severe visual impairment. Retinal artery occlusion (RAO) results from retinal thromboembolic events originating in the ipsilateral carotid artery, aortic arch, or cardiac origin^5^; retinal vein occlusion (RVO) is associated with thromboembolism due to degeneration of vascular walls and compression or vasospasm^6^. Retinal vascular occlusion is not only associated with predisposing factors for atheroembolic diseases^7^, but also with the risk of stroke, myocardial infarction, and total mortality^8^.

In terms of the safety in patients taking anticoagulants, intraocular bleeding is not a life-threatening ‘major’ complication, but is critical and can lead to severe visual loss and decrease vision-related quality of life. Previous studies have reported that there is an increased risk of intraocular bleeding with warfarin or other oral antithrombotics, raising concerns over the safety of these medications^9^. Hence, several studies on ocular diseases have examined the impact of anticoagulant therapy with NOAC, with a focus on ocular bleeding as a major safety issue^9–11^. While NOACs benefit patients with stroke and systemic thromboembolisms^12^, their efficacy on microvascular conditions, such as retinal vascular occlusion, requires further evaluation.

This study is to verify the efficacy and safety of NOACs in retinal disorders in terms of the incidence of retinal vascular occlusion and intraocular bleeding. We compared these incidences in NOAC and warfarin users by using a Korean cohort study.

## Results

**Figure 1:**
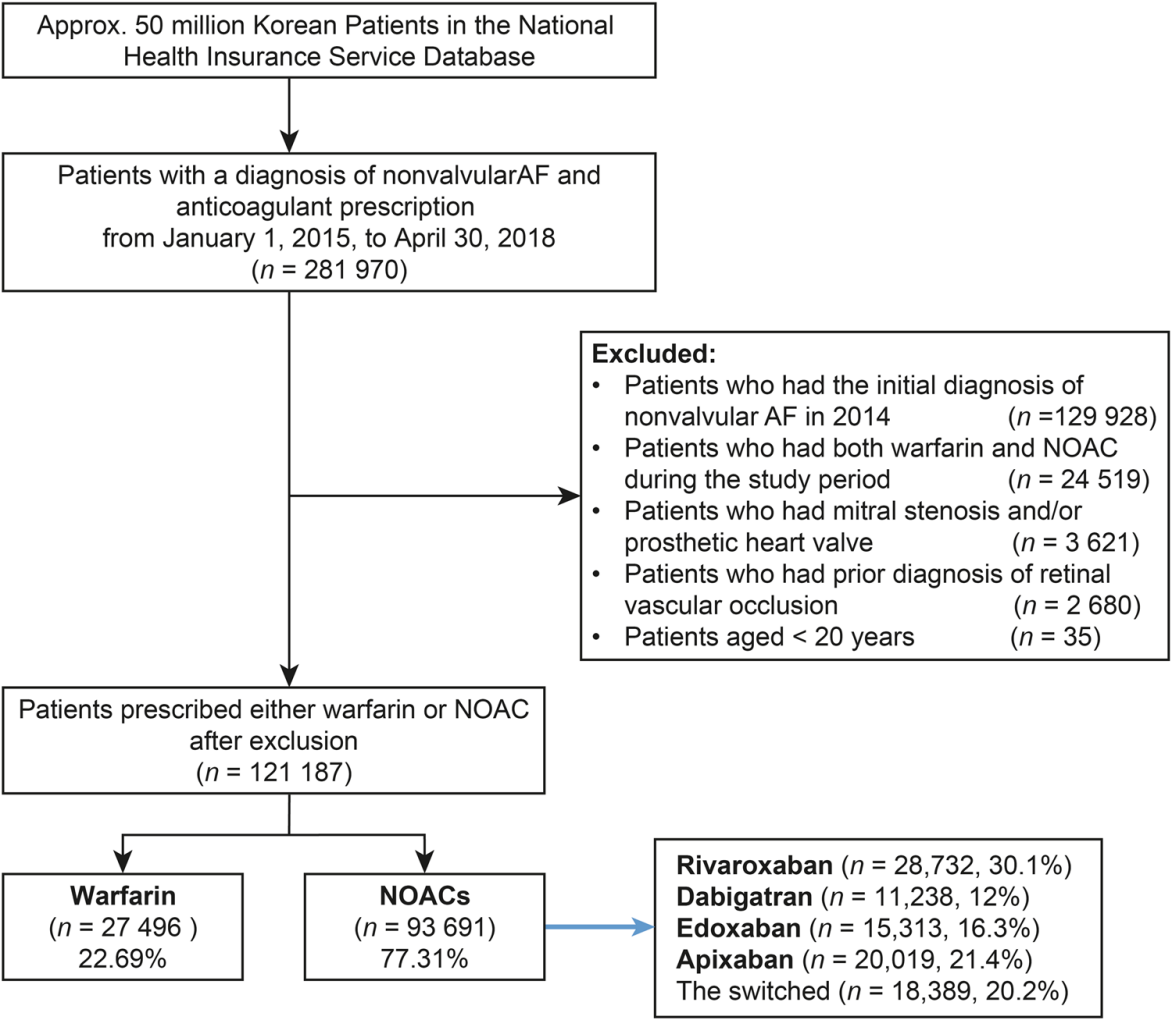
The flow chart of the study. AF: atrial fibrillation; NOAC: non-vitamin K antagonist oral anticoagulant.

The patients were initially categorized according to anticoagulant treatment. Among patients with non-valvular AF, 93,691 used NOAC and 27,496 used warfarin (Fig. 1). The NOAC users were categorized according to medications: dabigatran (n = 11,238; 12.0%), rivaroxaban (n = 28,732; 30.1%), apixaban (n = 20,019; 21.4%), edoxaban (n = 15,313; 16.3%), and those with switched NOACs (n = 18,389; 20.2%).

The average follow-up period was 2.68 ± 1.33 years for warfarin users and 1.24 ± 0.84 years for NOAC users. The baseline characteristics are listed in Table 1. The NOAC users were older, included more females, had more comorbidities, and had a higher CHA_2_DS_2_-VASc score. Among NOAC users, CHA_2_DS_2_-VASc score was highest with edoxaban (3.26), and lowest with apixaban (2.66).

**Table 1:** Baseline characteristics of included patients.

	Warfarin (n = 27,496)	NOAC (n = 93,691)	*P* value
Age at diagnosis of atrial fibrillation, years	66.39 ± 0.08	72.48 ± 0.03	<0.0001
Men, n (%)	16,002 (58.20)	49,333 (52.66)	<0.0001
CHA_2_DS_2_-VASc score^*^	2.66 ± 1.80	3.18 ± 1.61	<0.0001
Dabigatran		3.04 ± 1.57	
Rivaroxaban		3.05 ± 1.66	
Apixaban		2.66 ± 1.80	
Edoxaban		3.26 ± 1.62	
Comorbidities, n (%)^*^			
Hypertension	13,468 (48.98)	58,507 (62.45)	<0.0001
Dyslipidemia	6,643 (24.16)	26,181 (27.94)	<0.0001
Diabetes mellitus	6,788 (24.69)	25,081 (26.77)	<0.0001
Coronary heart disease	703 (2.56)	1,756 (1.87)	<0.0001
Stroke	4,433 (16.12)	13,227 (14.12)	<0.0001
Venous thromboembolism^†^	942 (3.43)	2,802 (2.99)	0.0002
Peripheral artery occlusive disease	2,152 (7.83)	9,309 (9.94)	<0.0001
Chronic kidney disease	2,626 (9.55)	1,829 (1.95)	<0.0001
Congestive heart failure	4,009 (14.58)	9,751 (10.41)	<0.0001
Medication, n (%)^*^			
Aspirin	5,745 (20.89)	24,315 (25.95)	<0.0001
P2Y12 inhibitor	4,072 (14.81)	15,619 (16.67)	<0.0001
Statin	9,747 (35.45)	35,131 (37.50)	<0.0001
NSAIDs	5,885 (21.40)	23,098 (24.65)	<0.0001
Index year, n (%)			<0.0001
2015	12,324 (44.82)	18,729 (19.99)	
2016	7,756 (28.21)	30,001 (32.02)	
2017	5,629 (20.47)	33,369 (35.62)	
2018	1,787 (6.5)	11,592 (12.37)	

^*^Calculated within wash-out period, 1 year before the first diagnosis date of atrial fibrillation.

^†^Venous thromboembolism included pulmonary thromboembolism and deep vein thromboembolism.

### Retinal vascular occlusion

**Figure 2:**
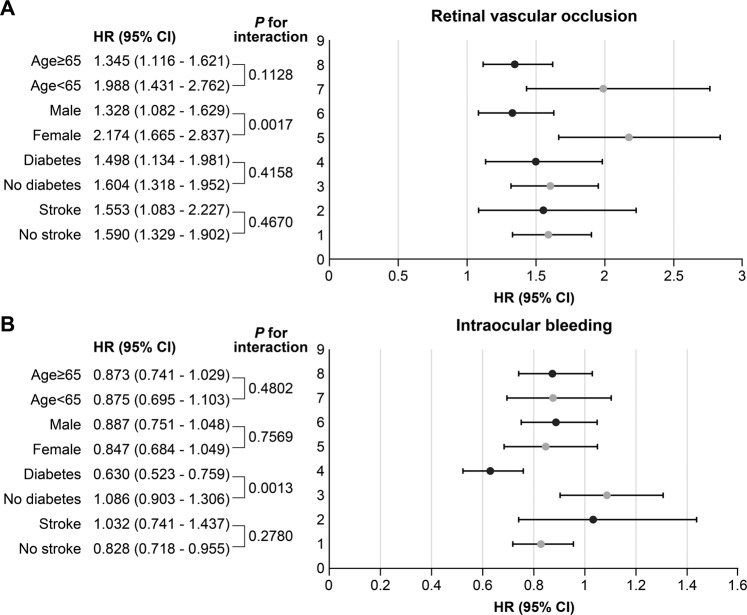
Subgroup analysis of HRs for retinal vascular occlusion (**A**) and intraocular bleeding (**B**) in NOACs users and warfarin users.

The incidence of retinal vascular occlusion was 0.91% (250/27,496) for warfarin users and 0.82% (766/93,691) for NOAC users. Person-years were taken into account as the follow-up period differed by patient, and the NOAC users showed higher incidence rate (IR) compared to warfarin users (IR 6.03 vs. 3.33 per 1000 person-years). The NOAC group had a higher risk of retinal vascular occlusion than the warfarin group (hazard ratio (HR), 1.61; 95% confidence interval (CI) 1.37–1.91), which was consistent after adjusting multiple covariates (Table 2). In a subgroup analysis according to age, sex, diabetes, and stroke, all the HRs for retinal vascular occlusion were higher and statistically significant in the NOAC group than those in warfarin group (Fig. 2A). Female patients had a higher HR for retinal vascular occlusion than male patients (HR 2.174 vs. 1.328, *P* for interaction = 0.0017). Among the different types of NOAC, when compared with warfarin, the HR for retinal vascular occlusion was 1.49, 1.47, 1.66, and 1.64 for dabigatran, rivaroxaban, apixaban, and edoxaban, respectively (Supplementary Table 1).

**Table 2:** Hazard ratios for retinal vascular occlusion and intraocular bleeding by type of anticoagulants.

Ocular disease	Drugs	Person-years	No. of cases	Event rate per 1,000 person-years	Unadjusted	Adjusted 1^*^	Adjusted 2^†^
					HR (95% CI)	HR (95% CI)	HR (95% CI)
Retinal vascular occlusion	Warfarin	75,026	250	3.33 (2.94, 3.77)	1.00	1.00	1.00
	NOAC	126,951	766	6.03 (5.62, 6.47)	1.59 (1.35,1.86)	1.54 (1.31, 1,82)	1.61 (1.37, 1.91)
RVO	Warfarin	75,166	155	2.06 (1.76, 2.41)	1.00	1.00	1.00
	NOAC	127,341	445	3.50 (3.18, 3.83)	1.69 (1.39,2.06)	1.65 (1.35, 2.02)	1.80 (1.45, 2.22)
RAO	Warfarin	75.303	40	0.53 (0.38, 0.72)	1.00	1.00	1.00
	NOAC	127,659	102	0.80 (0.66, 0.97)	1.41 (0.95,2.10)	1.35 (0.89, 2.04)	1.45 (0.96, 2.20)
Intraocular bleeding	Warfarin	74,674	402	8.43 (7.64, 9.29)	1.00	1.00	1.00
	NOAC	127,021	716	5.68 (5.24, 6.06)	0.86 (0.75,0.98)	0.89 (0.78, 1.02)	1.00 (0.87, 1.15)

CI, confidence interval; HR, hazard ratio; RAO, retinal artery occlusion; RVO, retinal vein occlusion.

^*^Adjusted for sex and age. ^†^Adjusted for sex, age, hypertension, dyslipidemia, chronic kidney disease, diabetes mellitus, coronary heart disease, stroke, venous thromboembolism, chronic kidney disease, congestive heart failure, the CHA_2_DS_2_-VASc score, and calendar index year.

Interestingly, the HR was found to be significantly higher for RVO (IRs 3.50 vs 2.06; HR 1.69; 95% CI 1.39–2.06) but not for RAO with NOAC (IRs 0.80 vs 0.53; HR 1.41; 95% CI 0.95–2.10). These were also consistent after adjusting for multiple covariates (Table 2).

**Figure 3:**
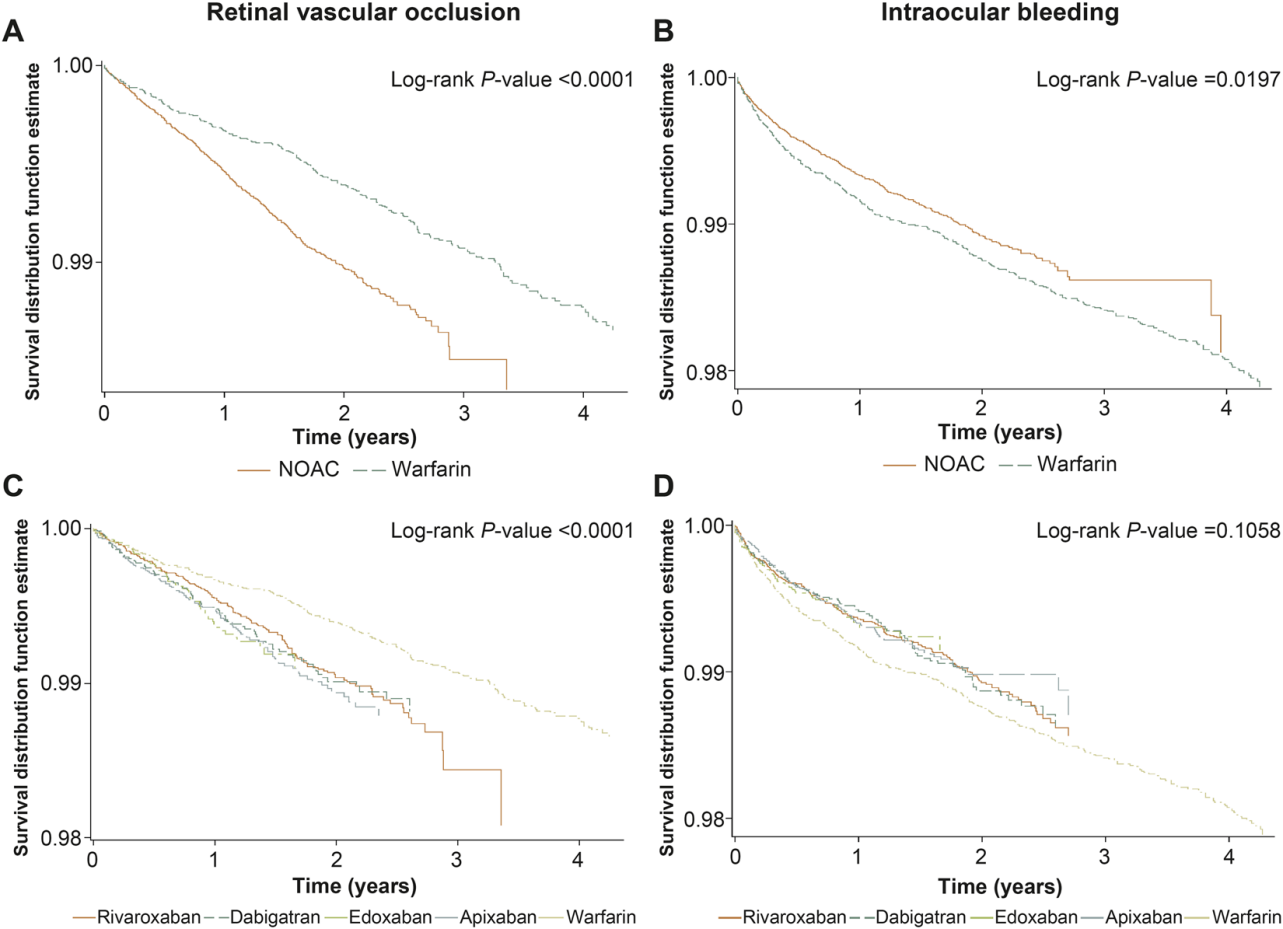
The Kaplan-Meier survival curves for retinal vascular occlusion (**A**) and intraocular bleeding (**B**) in NOACs users and warfarin users; for retinal vascular occlusion (**C**) and intraocular bleeding **(D**) among the different types of NOAC and warfarin.

The time to retinal vascular occlusion events with warfarin therapy was longer than with NOAC therapy (log-rank *P* value < 0.0001, Fig. 3A,C). The cumulative incidences also showed a higher rate of retinal vascular occlusion in the NOAC group than that in the warfarin group (Supplementary Fig. S1A,C).

**Figure 4:**
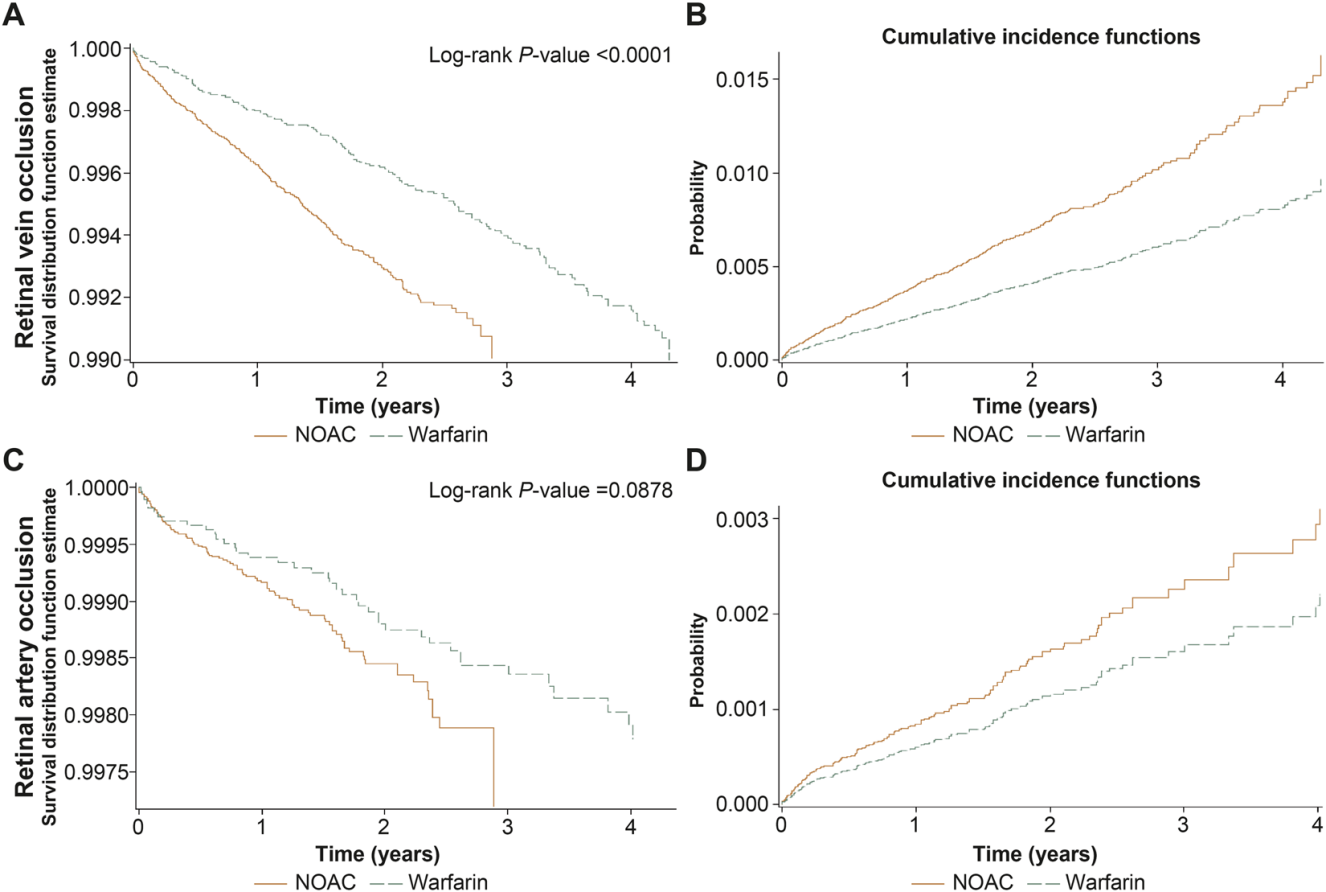
The Kaplan-Meier survival curves and cumulative incidences of retinal vein occlusion (**A,B**) and retinal artery occlusion (**C,D**) in NOAC users and warfarin users.

The Kaplan-Meier survival curves and cumulative risks are presented in Fig. 4, showing that the time to RVO events with warfarin therapy was longer than with NOAC therapy (log-rank *P* value < 0.0001, Fig. 4A), while this was not significant with RAO events (log-rank *P* value = 0.0878, Fig. 4C).

### Intraocular bleeding

In patients with underlying retinal vascular disorders related to intraocular bleeding, more patients with diabetic retinopathy were administered warfarin than NOAC (43.8% vs 34.4%, *P* < 0.0001). There were no differences for other retinal diseases, including age-related macular degeneration, choroidal neovascularization, and retinal vascular occlusion.

The patients administered NOACs had a lower risk for intraocular bleeding than warfarin users (IRs 5.68 vs. 8.43 per 1000 person-years; HR, 0.83; 95% CI 0.75–0.98, Table 2). This was consistent after adjusting for multiple covariates, but was not statistically significant. All the NOACs showed similar IR as well as HRs for intraocular bleeding when compared to warfarin (Supplementary Table 1). In a subgroup analysis according to age, sex, diabetes, or stroke, NOAC users had a lower risk for intraocular bleeding than warfarin users in patients with diabetes and those without a history of stroke (Fig. 2B). Especially, diabetic patients showed a lower HR for intraocular bleeding than patients without diabetes (HR 0.630 vs. 1.086, *P* for interaction = 0.0017). Among the different types of NOAC, the HR of intraocular bleeding was 0.84, 0.85, 0.80, and 0.81 for dabigatran, rivaroxaban, apixaban, and edoxaban respectively when compared with warfarin (Supplementary Table 1).

The survival rate for intraocular bleeding of NOAC users was greater than that of the warfarin users (log-rank *P* value = 0.0197, Fig. 3B). The survival for intraocular bleeding rate of every NOACs users was also greater than that of warfarin users, without statistical significance (log-rank *P* value = 0.1058, Fig. 3D). The cumulative incidences showed a lower rate of intraocular bleeding in the NOACs users than that in warfarin users (Supplementary Fig. S1B,D).

## Discussion

To the best of our knowledge, this is the first population-based cohort study to compare the efficacy of NOAC with warfarin for microvascular diseases in patients with non-valvular AF. Patients on NOAC had a higher risk of retinal vascular occlusion than those on warfarin. In terms of intraocular bleeding, this study found a lower risk of intraocular bleeding in NOAC users than in warfarin users.

NOACs are known to be non-inferior or even superior to warfarin for prevention of stroke and systemic embolism in patients with non-valvular AF, and also associated with lower rates of bleeding, especially life-threatening hemorrhage (*e.g*., intracranial bleeding)^3,13^. A cohort study reported that the incidence of intraocular bleeding was less in NOAC (dabigatran and rivaroxaban) users compared to that of warfarin users^9^. Other meta-analyses also revealed a lower risk of intraocular bleeding with NOAC use^14,15^, suggesting the safety of NOAC for intraocular bleeding.

Our study also showed that NOAC users have a lower association with intraocular bleeding than did warfarin users. The mechanism for lower bleeding risk is not fully understood, but several points suggested in studies on major bleeding might be applied. Warfarin inhibits vitamin K-dependent proteins (factors II, VII, IX, and X), thereby suppressing production of factor VIIa and formation of tissue factor-VIIa complexes^16^. The retinal pigment epithelial cells also express various coagulation factors including tissue factor and factor VII^17^, which may explain the safety of NOACs in terms of intraocular bleeding. NOACs directly inhibit specific coagulation factors (dabigatran: factor II; rivaroxaban, apixaban, and edoxaban: factor X), so that NOACs may preserve hemostatic mechanisms by selectively targeting thrombin and not interfering tissue factor-VIIa complexes^4,16^. Warfarin has limitations related with the diet and drug interaction and the need for anticoagulation monitoring, and the pharmacokinetics of warfarin is less predictable due to the polymorphisms in the genes (CYP2C9, VKORC1, etc.)^18,19^. Accordingly, the lower risk of bleeding with NOACs may be associated with the selective actions on coagulation pathway compared to warfarin^3^.

Retinal vascular occlusion is often associated with cardiovascular disease and have many of the same risk factors^8,20,21^. There are also cohort studies which have reported that retinal vascular occlusion increases the risk of stroke^8,22–24^. Interestingly, our study found that the risk of retinal vascular occlusion was higher in NOAC users than in warfarin users in Korea. Although this study did not investigate mechanisms associated with the higher risk of retinal vascular occlusion in NOAC users, the selective inhibition of specific coagulation factors by NOACs might be responsible^4^. NOACs are safe in terms of bleeding complications by preserving hemostatic mechanism^3^, while this would work the other way around as more retinal vascular occlusion in NOAC users. The lower trough level of NOACs compared to that of warfarin might be another factor for the inefficacy of NOACs^25^. It should be noted that patients with newly diagnosed retinal vascular occlusions are often referred to internists in clinical practice, who then evaluate systemic risk factors for future cardiovascular events and often prescribe anticoagulants^21,26^. However, there is a lack of high-quality evidence to support the routine use of anticoagulants or antithrombotic drugs for retinal vascular occlusions, and the benefits and risks of this therapy need to be considered in clinical settings^27^. Based on our findings, the appropriate anticoagulant should be selected in each patient based on the risk of intraocular bleeding and retinal vascular occlusions.

The risk of RAO was comparable between the NOAC and warfarin groups, while the risk of RVO was higher in the NOAC group compared to the warfarin group. There are possible reasons for this difference in efficacy of NOACs, besides the pharmacokinetics of NOACs acting on specific factors in coagulation pathway. First, RAO is related with atherosclerotic thromboembolism similar to stroke, and retinal artery emboli are commonly associated with AF, hypertension, and coronary artery disease^28^. On the other hand, the precipitating factors for RVO are more variable: vasospasm, inflammation, compression, and localized thrombosis^6^. Typical atherosclerosis risk factors are commonly associated with all types of RVO, while hyperviscosity and hypercoagulability are also related risk factors^26^. Second, RAO is relatively uncommon as compared to RVO^5,20^, which may affect statistical significance. Finally, the exact doses for anticoagulants were not investigated in this study. There is a tendency in Asian countries to use the lower doses of NOACs in clinical practice^2,29,30^, which may have affected the results of this study.

There were some interesting points in subgroup analyses. Diabetic patients had a remarkably lower risk of intraocular bleeding with NOACs than the opposite group. Diabetes is a well-known risk factor for ischemic stroke, while there were controversies on hemorrhagic risk associated with NOAC treatment in diabetic patients with AF^31^. Most studies showed comparable major bleeding rate in patients without significant interaction with diabetes^31^, while there was also a report that diabetic patients showed a reduced rate of intracranial hemorrhage when treated with dabigatran than with warfarin^32^. The mechanisms for this lower risk of bleeding in diabetic patients are not clarified, but less diet and drug interactions with NOACs might explain one of possible reasons for the safety of NOACs in diabetic patients^3^. In terms of retinal vascular occlusion, female patients showed a higher risk of retinal vascular occlusion with NOACs than male patients. Our study did not investigate the dosage by sex, while more female patients are known to be prescribed lower dose of NOACs in Korea^30^. This may result in a higher risk of retinal vascular occlusion with lower efficacy. Similarly, off-label low dose of NOAC was associated with increased risk of cerebral ischemic events^33^, and these tendencies of ischemic event need further investigation along with the gender difference.

There was no definite superiority among the different type of NOACs. All the NOACs showed a higher risk for retinal vascular occlusion and a comparable risk of intraocular bleeding than did warfarin. Regarding ocular safety in the present study, *i.e*. intraocular bleeding, all the NOACs showed a lower risk of bleeding than did warfarin. This is similar to previous cohort studies which reported a reduced risk for intraocular bleeding^9,15^. Few guidelines have addressed the issue of anticoagulant use for treatment of retinal vascular occlusions and the possible risk for intraocular bleeding^21,26^. No specific medication has been established to directly improve perfusion, and current ophthalmic treatments focus on the complications of retinal vascular occlusions such as macular edema, ocular neovascularization, and intraocular bleeding^6,7^. All NOACs have a merit in terms of safety for intraocular bleeding; however, efficacy for the prevention of retinal vascular occlusion was not clarified in this study. As the incidence and prevalence of AF increases with advancing age, further studies are needed to investigate the efficacy of NOACs for preventing microvascular disease.

This cohort study has some limitations. First, the presence of retinal vascular occlusion was defined by the presence of diagnostic codes from HIRA database. Although the diagnostic codes are mandatory for any patient, the accuracy of diagnoses was not confirmed by the medical records. However, this limitation might affect both NOAC and warfarin groups and therefore, a one-sided application might be prevented. Second, we could not investigate the difference between standard-dose NOAC users and low-dose NOAC users. As mentioned above, Asia shows a tendency for prescribing lower doses than do Western countries^15,29^. We could not classify the patients according to dose as many patients changed the dose during the study period. Third, detailed information on patients’ history of socioeconomic status, smoking, or alcohol consumption, and laboratory profiles including international normalized ratio (INR) of prothrombin time were not available from the HIRA database. Some of the patients on warfarin could be in supratherapeutic range of INR, but this is less likely as the proportion of warfarin users in supratherapeutic range is lower than those in subtherapeutic range in Asia^34^. Although we adjusted for various confounding factors using diagnostic and procedure codes from the HIRA database, more studies in real clinical practice are needed to confirm the effects of NOACs in retinal microvascular diseases. Lastly, the findings in this study need cautious interpretation due to the retrospective nature. Although the association of NOACs and higher incidence rate of retinal vascular occlusion was found in this study, further studies prospective in nature are needed to verify this association.

In conclusion, the present study showed that use of a NOAC was not superior to warfarin in terms of retinal vascular occlusions, unlike its effect on the treatment of stroke or systemic embolism. However, NOACs were safe in terms of intraocular bleeding. Further studies in clinical practice may help to identify the most appropriate anticoagulants for patients with microvascular diseases.

## Methods

### Study design and participants

This retrospective cohort study protocol was reviewed and approved by the Institutional Review Board of Ajou University Hospital (AJIRB-MED-EXP-18-380). The requirement for informed consent was waived by the Institutional Review Board of Ajou University Hospital as the data in this database were de-identified. All methods were performed in accordance with relevant guidelines and regulations.

The Health Insurance Review and Assessment (HIRA) service reviews all of the health claims in Korea, including those submitted through the Korean National Health Insurance service which covers 97% of the population. Diagnoses are coded using the International Classification of Diseases, 10^th^ revision (ICD-10). The inclusion criteria were: (i) patients aged ≥20 years, (ii) ≥ 1 criteria for non-valvular AF (I48.XX, ICD-10 codes) in the primary or within 3rd order of secondary diagnosis, and (iii) ≥1 prescription of NOACs or warfarin between January 2015 and April 2018. The exclusion criteria were patients who had: (i) been diagnosed with AF in 2014 to select the patients who were newly diagnosed with AF from 2015, (ii) both warfarin and NOAC during the study period, (iii) mitral stenosis and/or prosthetic heart valve, (iv) prior diagnosis of retinal vascular occlusion, and (v) aged <20 years at the time of AF diagnosis. Those who were diagnosed with AF prior to 2014 but received a prescription between January 2015 and April 2018 were excluded. These criteria are summarized in Fig. 1.

### Outcomes and covariates

Each participant’s index date was set to the date of earliest prescription. The follow-up for each event extended from the index date to the earliest incidence of retinal vascular occlusion, or intraocular bleeding, or the end of the observation date (April 30, 2018), if events did not occur.

The presence of retinal vascular occlusion was determined on the basis of diagnostic codes of retinal vascular occlusions in the primary or within 3rd order of secondary diagnosis, including RAO (H34.0, H34.1, and H34.2; ICD-10), RVO (H34.8, ICD-10), and other retinal vascular occlusions (H34.9, ICD-10). Similarly, the presence of intraocular bleeding was determined using the diagnostic codes for non-traumatic hyphema (H21.0, ICD-10), vitreous hemorrhage (H43.1 and H45.0, ICD-10), retinal hemorrhage (H35.6, ICD-10), and choroidal hemorrhage (H31.3). For retinal disorders leading to intraocular bleeding, the presence of the previously stated retinal vascular diseases was investigated. The NOACs included those available in Korea and covered by the national insurance service such as dabigatran, rivaroxaban, apixaban, and edoxaban.

The following covariates were included for baseline adjustments to minimize confounding factors: age at diagnosis of AF, sex, hypertension, dyslipidemia, chronic kidney disease, diabetes mellitus, coronary heart disease, stroke, venous thromboembolism, CHA_2_DS_2_-VASc score, and calendar index year. The CHA_2_DS_2_-VASc score, which is used in guidelines for stroke prevention in AF implying comorbid diseases (congestive heart failure, hypertension, age, diabetes mellitus, prior stroke of transient ischemic attack, vascular disease, and female), was used in this study including AF patients^35^. A list of variables with corresponding ICD-10 codes, procedures, operation codes, and anatomical therapeutic chemical codes is provided in Supplementary Table S2.

### Statistical analysis

The baseline characteristics were summarized and compared for the patients with NOAC and warfarin using a chi-square test of homogeneity for categorical variables and a two sample *t*-test for continuous variables.

The IRs were stratified by each medication and estimated with the number of retinal vascular occlusion or intraocular bleeding events per 1,000 person-years, and the 95% CI was calculated by the Mid-P exact test. The cox proportional-hazards regression model was used to calculate the HR and 95% CI. The HR with 95% CI was presented with separate statistical models; (i) unadjusted, (ii) adjusted with age and sex, and (iii) adjusted with age, sex, CHA_2_DS_2_-VASc score, calendar index year, and comorbidities (hypertension, dyslipidemia, chronic kidney disease, diabetes mellitus, coronary heart disease, stroke, and venous thromboembolism). The time from initiation of NOAC or warfarin to primary events was assessed using the Kaplan-Meier survival curve and compared using the log-rank test. Additionally, the unadjusted cumulative incidence was calculated according to the type of medication.

In the secondary analysis, the same analysis was performed with different types of NOACs, such as dabigatran, rivaroxaban, apixaban, and edoxaban, and compared with warfarin. Those who switched medications (within the NOAC group) at any point in the time were excluded in the secondary analysis. Additionally, the unadjusted HR was presented by subgroups according to age ≥65, sex, diabetes, and stroke. All statistical analyses were performed using SAS software (version 9.4, SAS Institute Inc., Cary, NC, USA). A *P* value of <0.05 was considered significant.

## Supplementary information

Supplementary information.
