# Supplementary material for: Efficacy and safety of non-vitamin K-antagonist oral anticoagulants for retinal vascular diseases in patients with atrial fibrillation: Korean cohort study
Source: Sci Rep. 2020 Mar 12;10:4577. doi: 10.1038/s41598-020-61609-8 (PMC7067845; doi:10.1038/s41598-020-61609-8)
Supplement: Supplementary file 1 — Supplementary information. [file 41598_2020_61609_MOESM1_ESM.pdf]

Efficacy and safety of non-vitamin K-antagonist oral anticoagulants for retinal vascular diseases in patients with atrial fibrillation: Korean cohort study

Se-Jun Park[1†], Eunyoung Lee[2,3†], Kihwang Lee[4], Bumhee Park[2,3*], Yoo-Ri Chung[4*]

[1]Division of Cardiology, Department of Internal Medicine, Gangneung Asan Hospital, University of Ulsan College of Medicine, Gangneung, Korea

[2]Department of Biomedical Informatics, Ajou University School of Medicine, Suwon, Korea

[3]Office of Biostatistics, Ajou Research Institute for Innovative Medicine, Ajou University School of Medicine, Suwon, Korea

[4]Department of Ophthalmology, Ajou University School of Medicine, Suwon, Korea

[†]These authors equally contributed to this work.

[*]These are co-corresponding authors.

SUPPLEMENTAL INFORMATION

Table S1. Hazard ratios for retinal vascular occlusion and intraocular bleeding among the different types of NOAC

Ocular disease	Drugs	Person-years	No. of cases	Event rate per 1,000 person-years	Unadjusted	Adjusted 1*	Adjusted 2†
					HR (95% CI)	HR (95% CI)	HR (95% CI)
Retinal vascular occlusion	Warfarin	75,026	250	3.33 (2.94, 3.77)	1.00	1.00	1.00
	Dabigatran	17,486	99	5.66 (4.63, 6.86)	1.49 (1.15,1.94)	1.44 (1.11, 1.87)	1.49 (1.14, 1.94)
	Rivaroxaban	42,268	235	5.56 (4.88, 6.31)	1.47 (1.20,1.80)	1.44 (1.17, 1.77)	1.51 (1.22, 1.86)
	Apixaban	24,228	148	6.11 (5.18, 7.16)	1.66 (1.32,2.09)	1.61 (1.28, 2.03)	1.69 (1.33, 2.14)
	Edoxaban	12,733	97	7.62 (6.21, 9.25)	1.64 (1.23,2.20)	1.60 (1.19, 2.14)	1.74 (1.29, 2.36)
Intraocular bleeding	Warfarin	74,674	402	8.43 (7.64, 9.29)	1.00	1.00	1.00
	Dabigatran	17,492	94	5.37 (4.37, 6.55)	0.84 (0.67,1.06)	0.85 (0.67, 1.07)	0.96 (0.76, 1.22)
	Rivaroxaban	42,271	230	5.44 (4.77, 6.18)	0.85 (0.72,1.00)	0.87 (0.73, 1.03)	1.01 (0.84, 1.20)
	Apixaban	24,264	131	5.40 (4.53, 6.39)	0.80 (0.65,0.98)	0.82 (0.67, 1.01)	0.92 (0.75, 1.14)
	Edoxaban	12,755	79	6.19 (4.94, 7.68)	0.81 (0.63,1.04)	0.83 (0.64, 1.07)	1.00 (0.77, 1.30)

CI, confidence interval; HR, hazard ratio.

*Adjusted for sex and age.

†Adjusted for sex, age, hypertension, dyslipidemia, chronic kidney disease, diabetes mellitus, coronary heart disease, stroke, systemic embolism, chronic kidney disease, congestive heart failure, the CHA_2DS_2-VASc score, and calendar index year.

Table S2. List of diagnoses, treatments, and procedures, and their corresponding codes

Variables	Codes
**Diagnoses**	
Hypertension	I10-I13
Dyslipidemia	E78.0, E78.1, E78.2, E78.4, E78.5, E78.8, E78.9
Atrial fibrillation	I48.0, I48.1, I48.2, I48.3, I48.4, I48.9
Diabetes mellitus	E10-E14
Coronary heart disease	I21, I22, I25.2
Heart failure	I50
Stroke	I60-I66, G45
Deep vein thrombosis	I80.2
Pulmonary thromboembolism	I26.0, I26.9A, I26.9B, I26.9H, I26.9J, I26.9K
Peripheral artery disease	I70.2-3, I70.9, I73.1, I73.8-9
Chronic kidney disease	N18
Aorta plaque	I70.0, I70.8
Retinal vascular occlusion	
Retinal vein occlusion	H34.8
Retinal artery occlusion	H34.0, H34.1, H34.2
Other retinal vascular occlusion	H34.9
Diabetic retinopathy	H36.0
Age-related macular degeneration	H35.31B, H35.39
Choroidal neovascularization	H31.8
**Medications**	
NOAC	
Rivaroxaban	511401ATB, 511402ATB, 511403ATB, 511404ATB
Dabigatran	613701ACH, 613702ACH, 613703ACH
Edoxaban	643601ATB, 643602ATB, 643603ATB
Apixaban	617001ATB, 617002ATB
Warfarin	249101ATB, 249102ATB, 249103ATB, 249104ATB, 249105ATB, 249106ATB, 249107ATB, 249108ATB, 249109ATB,

Low dose acetylic salicylic acid	111001ATE, 111002ATE, 111003ATE
Receptor P2Y12 antagonists	136901ATB, 239201ATB, 597301ATB, 597302ATB, 615901ATB, 615902ATB
NSAID	142301ATB, 142301ATR, 142302ATB, 142303ATB, 199401ATR, 199404ATE, 199501ATB, 199502ATB
Statin	
Atorvastatin	111501ATB, 111502ATB, 111503ATB, 111504ATB
Fluvastatin	162401ATB, 162402ATB, 161403ATR
Pitavastatin	470901ATB, 470902ATB, 470903ATB
Lovastatin	185801ATB
Pravastatin	216601ATB, 216602ATB, 216603ATB, 216604ATB
Simvastatin	227801ATB, 227802ATB, 227803ATB, 227806ATB
Rosuvastatin	454001ATB, 454002ATB, 454003ATB

NOAC: non-vitamin K oral anticoagulant; NSAID: non-steroidal anti-inflammatory drug.

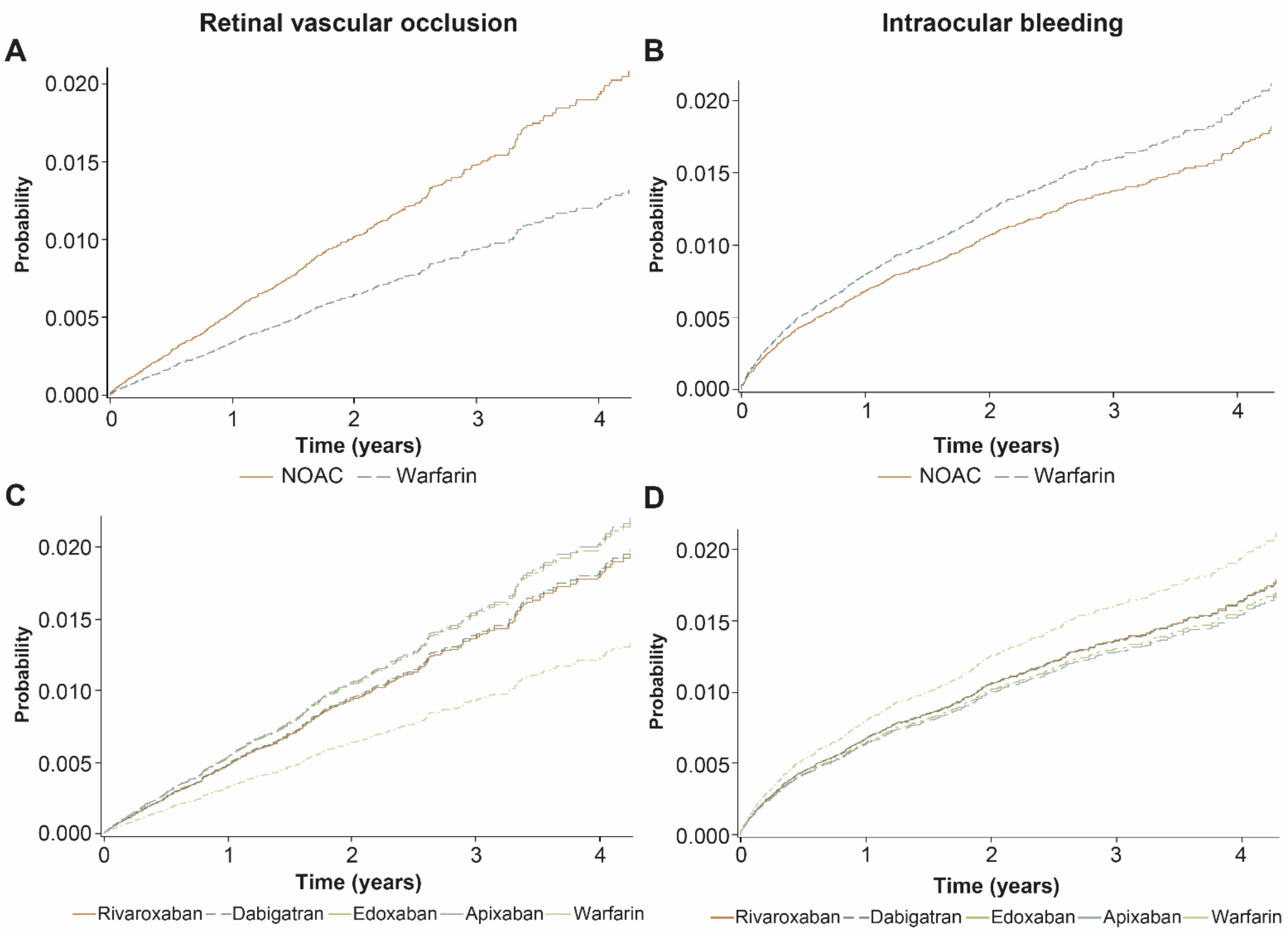

Figure S1. Cumulative incidence for retinal vascular occlusion (A) and intraocular bleeding (B) in NOAC users and warfarin users; for retinal vascular occlusion (C) and intraocular bleeding (D) among different types of NOAC and warfarin.
